# The Role Played by Wnt/β-Catenin Signaling Pathway in Acute Lymphoblastic Leukemia

**DOI:** 10.3390/ijms21031098

**Published:** 2020-02-07

**Authors:** Francesca Chiarini, Francesca Paganelli, Alberto M. Martelli, Camilla Evangelisti

**Affiliations:** 1 CNR Institute of Molecular Genetics “Luigi Luca Cavalli-Sforza”, Unit of Bologna, 40136 Bologna, Italy; francesca.chiarini@cnr.it; 2 IRCCS Istituto Ortopedico Rizzoli, 40136 Bologna, Italy; 3 Department of Biomedical and Neuromotor Sciences, University of Bologna, 40126 Bologna, Italy; francesca.paganell16@unibo.it (F.P.); alberto.martelli@unibo.it (A.M.M.)

**Keywords:** Wnt/β-catenin, acute lymphoblastic leukemia, hematopoietic stem cells, leukemic stem cells, signaling pathway, targeted therapy

## Abstract

Acute lymphoblastic leukemia (ALL) is an aggressive hematologic neoplastic disorder that arises from the clonal expansion of transformed T-cell or B-cell precursors. Thanks to progress in chemotherapy protocols, ALL outcome has significantly improved. However, drug-resistance remains an unresolved issue in the treatment of ALL and toxic effects limit dose escalation of current chemotherapeutics. Therefore, the identification of novel targeted therapies to support conventional chemotherapy is required. The Wnt/β-catenin pathway is a conserved signaling axis involved in several physiological processes such as development, differentiation, and adult tissue homeostasis. As a result, deregulation of this cascade is closely related to initiation and progression of various types of cancers, including hematological malignancies. In particular, deregulation of this signaling network is involved in the transformation of healthy HSCs in leukemic stem cells (LSCs), as well as cancer cell multi-drug-resistance. This review highlights the recent findings on the role of Wnt/β-catenin in hematopoietic malignancies and provides information on the current status of Wnt/β-catenin inhibitors with respect to their therapeutic potential in the treatment of ALL.

## 1. Introduction

Acute lymphoblastic leukemia (ALL) is an aggressive hematological disorder that can originate from either B-lineage (B-ALL) or T-lineage (T-ALL) lymphoid precursors and is characterized by a marked heterogeneity at both molecular and clinical levels.

B-ALL is the most frequent neoplasia in childhood, which represents 80% of pediatric ALL [1]. Thanks to progress in chemotherapy protocols, the five-year survival rates are above 90% [2]. On the contrary, ALL has a much worse outcome in adults over 60 years of age where the survival rate is under 30% [3]. Hence, although chemotherapy regimens greatly improved the prognosis, a considerable percentage of patients does not respond and relapses.

T-ALL is caused by clonal transformation of T-lineage lymphoid precursors and comprises 10–15% of pediatric ALL cases and around 25% of adults [4]. T-ALL has a worse prognosis compared to B-ALL and, especially in adults, drug-resistance remains an unresolved issue in the treatment of T-ALL and dose escalation of current chemotherapeutics is limited by side effects. Hence, the identification of novel targeted therapies to support conventional chemotherapy is needed [5].

Recently, it has been demonstrated that several key drivers of cell growth, proliferation, survival, and differentiation networks contribute to the pathogenesis of ALL. This opens new opportunities for future innovative therapies that could ameliorate the prognosis of ALL patients, especially for the high-risk or relapsed ones [6].

The Wnt/β-catenin network is an evolutionarily conserved signaling pathway that plays a fundamental role in many physiological processes such as differentiation, proliferation, and cell fate determination. Hence, aberrant activation of this cascade may underlie the initiation and progression of various types of cancers, including hematologic malignancies [7].

In this review, the current insights into the relevance of Wnt/β-catenin signaling in hematopoiesis and ALL development will be highlighted. The pharmacological application of Wnt/β-catenin inhibitors in view of therapeutic strategies will also be described.

## 2. Wnt/β-Catenin Signaling Pathway

The Wnt/β-catenin pathway regulates several physiological processes such as embryogenic development, adult tissue homeostasis, wound healing, and stem cell maintenance by regulating the cell fate, differentiation, apoptosis, polarity, and migration [8].

The Wnt family is a group of secreted cysteine-rich glycoproteins (19 in humans) that carry out their functions through three different signaling pathways named β-catenin-independent, “non-canonical” β-catenin-dependent, and “canonical” β-catenin-dependent (hereinafter Wnt/β-catenin) which is the most studied [9].

**Figure 1:**
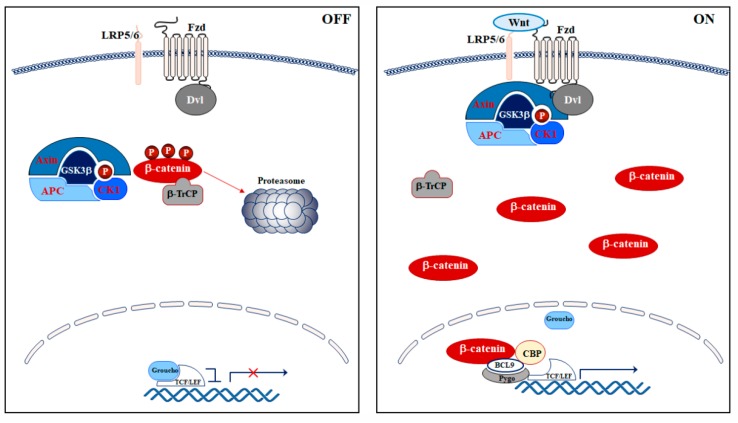
Schematic representation of the Wnt/β-catenin signaling pathway, which is inactive in the absence of Wnt ligands (OFF) and active upon binding of Wnt ligands (ON). See text for details of pathway activation. Arrows show activation while T-bars show inhibition.

In mammals, some consecutive steps characterize the Wnt/β-catenin axis: Wnt/β-catenin activation at the membrane level, its stabilization in the cytoplasm, where β-catenin can accumulate, and translocation of β-catenin to the nucleus where it activates Wnt/β-catenin-dependent target genes [8] (Figure 1).

The activation of the pathway occurs at the cellular membrane, where Wnt ligands bind to the seven transmembrane-domain protein receptors Frizzled (Fzd) and/or to the low-density lipoprotein receptor-related protein (LRP) 5/6. This interaction leads to the inhibition of the axin degradasome destruction complex, which is a multiprotein complex that controls the cytoplasmic amount of β-catenin via phosphorylation, and, thereby, triggers β-catenin degradation by the proteasome in the absence of Wnt [10]. The destruction complex comprises the tumor suppressor adenomatous polyposis coli (APC), the axin scaffold protein, and two Ser/Thr kinases: glycogen synthase kinase 3 (GSK3) β and casein kinase 1 (CK1). In the absence of Wnt ligands, CK1 phosphorylates β-catenin at Ser45 residue and GSK3β at Ser33/Ser37/Thr41 residues. Then, the β-transducin repeats-containing protein (β-TrCP), an E3-ubiquitin ligase, ubiquitinates phosphorylated β-catenin, which becomes a target for proteasomal degradation [10].

When Wnt binds to Fzd and/or LRP5/6 receptors, the Wnt/β-catenin pathway is activated and the axin degradasome is inhibited [9]. As a consequence, Dishevelled (Dvl) is activated and recruits the degradasome complex to the plasma membrane, and, thereby, promotes the interaction between LRP5/6 and axin [11,12]. Consequently, LRP5/6 is phosphorylated at specific amino acidic residues (Ser1490, Thr1530, Thr1572, Ser1590, Ser1607) [13], acting as a direct competitive inhibitor of GSK3β [14].

Moreover, inactivation of GSK3β through Akt-dependent Ser9 phosphorylation prevents the phosphorylation of β-catenin, which allows its stabilization and accumulation in the cytoplasm. Stabilized β-catenin translocates to the nucleus where it binds to transcription factors, notably T-cell factor (TCF) and lymphoid enhancing factor (LEF), TCF/LEF. This interaction displaces the co-TCF/LEF repressor Groucho, whose function under basal conditions is to compact chromatin [15]. Groucho and TCF/LEF form a multiprotein complex, which is also termed Wnt enhanceosome, that recruits transcriptional co-activators and histone modifiers such as the ATP-dependent helicase Brahma-related gene 1 (BRG1, also known as SMARCA4), cyclic adenosine mono phosphate response element (CREB)-binding protein (CBP), p300, B-cell lymphoma 9 (BCL9), and pygo [15,16]. The Wnt enhanceosome regulates chromatin remodeling and activates the transcription of β-catenin-dependent genes involved in cell growth and survival, including *C-MYC*, *CCND1*, *BIRC5*, and *CDKN1a* [9]. C-myc is a proto-oncogene that activates cyclin D1 and simultaneously inhibits p21 and p27, which leads to uncontrolled cell proliferation [17,18].

## 3. Wnt/β-Catenin Signaling Pathway Regulation

**Figure 2:**
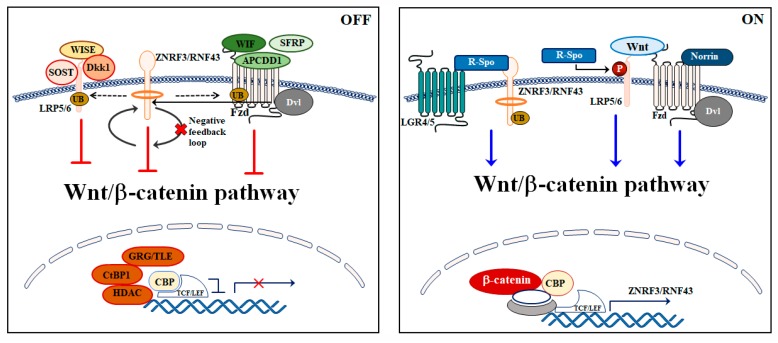
Schematic representation of the regulation of the Wnt/β-catenin signaling pathway when it is inactive (OFF) and when it is active (ON). OFF: ZNRF3 and RNF43 are transmembrane molecules that downregulate Wnt/β-catenin signaling. They promote the ubiquitination (UB) and lysosomal degradation of Fzd and LRP5/6. Secreted SFRP, APCDD1, and WIF can directly bind Fzd to prevent activation of receptors. Other Wnt antagonists, Dkk1 and Wise, inhibit by binding to the co-receptors LRP5/6. GRG/TLE, CtBP1, and HDAC negatively control Wnt/β-catenin pathway binding to TCF. ON: The Wnt agonists R-spondins interact on the cell surface with members of the LGR4/5 family to enhance Wnt signaling. Binding of R-spondin to ZNRF3/RNF43 inhibits ZNRF3, which enhances the Wnt/β-catenin pathway activity. Norrin acts by interacting with Fzd4 and requiring LRP5/6 for its activation. Arrows show activation while T-bars show inhibition.

Under physiological conditions, Wnt/β-catenin signaling pathway is strictly and efficiently regulated at many levels through multiple positive and negative feedback mechanisms (Figure 2).

R-spondins represent the main activators of Wnt/β-catenin axis. They are a family of secreted proteins that prevent LRP5/6 internalization and increase the activation of the Wnt/β-catenin cascade through a synergism with Wnt ligands. It has been shown that R-spondin 1 improves Wnt/β-catenin pathway activity by enhancing β-catenin stabilization and phosphorylation of LRP6 [19]. Importantly, these secreted proteins require leucine-rich repeat-containing G-protein coupled receptor (LGR) 4 and 5 to be active [20,21]. R-spondins act by counterbalancing the negative modulation of two homologues E3 ligases: the cell-surface transmembrane E3 ubiquitin ligase zinc and ring finger 3 (ZNRF3) and its homologue ring finger 43 (RNF43), which increases the membrane level of Wnt receptors [22,23].

ZNRF3 and RNF43 are single-pass transmembrane E3 ligases carrying intracellular RING domains. They act as powerful negative regulators of the Wnt/β-catenin pathway through their ability to promote the ubiquitination and lysosomal degradation of Fzd and LRP5/6. Of note, RNF43 and ZNRF3 are encoded by Wnt target genes, which leads to a negative feedback loop [22,23]. Loss of these two proteins causes hyper-responsiveness to endogenous Wnt signals and dysregulation of R-spondin/ZNRF3/RNF43 feedback loops have been identified in different types of cancer. In pancreatic ductal adenocarcinoma, loss-of-function mutations of RNF43 and ZNRF3 correlated with cancer development [24,25] while amplification of R-spondin genes was reported in more than 18% samples of patients affected by colorectal and endometrial cancer [26]. The overexpression of R-spondins seems also to be involved in the tumorigenesis process in colorectal carcinoma [27].

Norrin is an extracellular growth factor that represents another key activator of the Wnt/β-catenin pathway, which interacts with Fzd4 and requires LRP5/6 for its activation [28,29,30].

Several negative regulators finely tune the Wnt/β-catenin network via their binding to Wnt ligands. For instance, secreted Frizzled-related proteins (SFRPs), in concert with Wnt inhibitory factor (WIF) and adenomatosis polyposis down-regulated 1 (APCDD1), inhibit Wnt/β-catenin signaling by preventing Fzd and Wnt binding [31,32].

Sclerostin (SOST) and sclerostin domain containing 1 (SOSTDC1), alias WISE, counteract Wnt/β-catenin signaling by binding to LRP5/6 [33,34,35]. C-Terminal Binding Protein (CtBP) 1, histone deacetylases (HDAC)s, groucho/transducin-like enhancer (GRG/TLE), and the secreted glycoproteins Dickkopf family (Dkks) represent other important Wnt/β-catenin inhibitors [36,37]. GRG/TLE, CtBP1, and HDACs interact with nuclear TCF to turn off the transcription of Wnt target genes in the absence of nuclear β-catenin [38], whereas Dkks bind with high affinity to LRP5/6, and, thereby, prevents Wnt and LRP5/6 interaction [39]. While Dkk1 always acts as a Wnt/β-catenin inhibitor, Dkk2 may act as either an inhibitor or an activator depending on the cell context. In HEK293T and NIH3T3 cell lines, it has been demonstrated that, when co-transfected with Wnt and Fzd, Dkk2 acts as an activator, but when co-transfected with LRP5/6, it may act as inhibitor [40,41,42]. In Xenopus, the overexpression of Dkk2 strengthened the Wnt/β-catenin pathway by synergizing with co-expressed Fzd8 [40] or LRP6 [43].

Moreover, it has been demonstrated that the positive Wnt/β-catenin regulator Dvl also acts by recruiting ZNRF3 and RNF43 to Fzd receptors and, thus, inhibits the pathway [44]. The ZNRF3/RNF43 inhibition activity requires Dvl that acts as an adaptor for the E3 ligases.

## 4. Wnt/β-Catenin in Hematopoiesis

Hematopoiesis is a lifelong and tightly regulated process that gives rise to all blood cell types. Hematopoiesis depends on hematopoietic stem cells (HSCs), which is a pool of rare stem cells characterized by pluripotency and self-renewal through asymmetric division, quiescence, and multi-lineage differentiating potential. Even though dormancy is the preferred status for HSCs, upon specific stimuli, these cells can self-renew, and yield hematopoietic progenitors that, in turn, give rise to the mature hematopoietic cell lineages [45]. HSCs reside in the bone marrow (BM) niche, which is a specialized microenvironment that has a pivotal role in regulating the physiology of healthy HSCs.

Wnt/β-catenin network controls the delicate balance between self-renewal and lineage commitment of HSCs as well as the HSCs maintenance [46]. This tight regulation is very complex as it depends on different aspects that include the development stage, the local amount of Wnt proteins, and BM niche factors [47].

The specific roles played by Wnt/β-catenin axis on healthy HSCs are still a matter of debate as several studies, based on loss-of-function and gain-of-function approaches, resulted in conflicting results.

It has been shown that aberrant overexpression of β-catenin, in both in vitro and in vivo studies, activated the long-term growth of HSCs [47]. On the other hand, two independent groups showed that overexpressed β-catenin led to enforced HSCs cell cycle entry in mice. Thereby, it caused the exhaustion of the long-term HSCs pool [48] along with hematopoietic failure and loss of HSCs repopulating stem cell activity [49].

**Figure 3:**
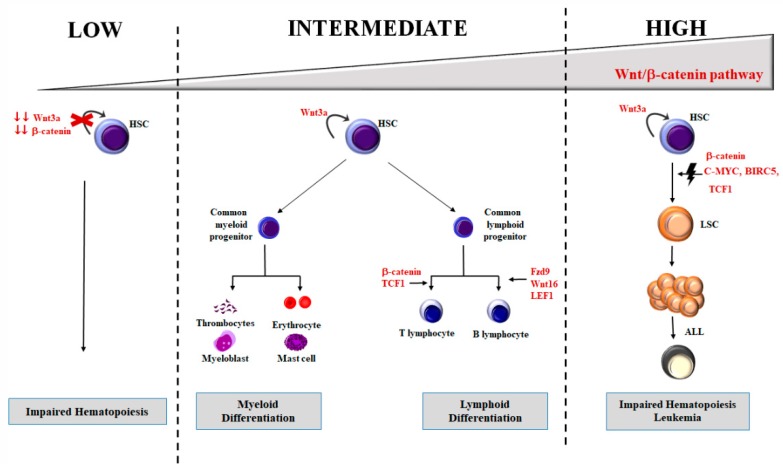
Role of Wnt/β-catenin signaling pathway in hematopoiesis and leukemogenesis. Wnt/β-catenin axis is differentially regulated during hematopoiesis. When the pathway is inhibited, correct hematopoiesis is compromised whereas, when slightly activated, there is an increase of hematological stem cell (HSC) clonogenicity and myeloid development. Intermediate-high levels lead to T-cell development. In addition, very high Wnt/β-catenin activation may lead to impaired hematopoiesis and leukemia development.

Luis and colleagues [50] gave an explanation for these conflicting data by hypothesizing that Wnt/β-catenin signaling might be differentially activated during hematopoiesis, while exerting its effects on HSCs in a concentration-dependent manner. When the Wnt/β-catenin network is mildly activated, HSCs may increase clonogenicity and myeloid development. Nevertheless, when it is over-activated, HSCs stemness is inhibited, which leads to an impairment of HSCs self-renewal and differentiation [50,51] (Figure 3).

Likewise, loss-of-function studies produced conflicting data. β-catenin-deficient mice displayed a decreased HSCs self-renewal capacity, long-term growth, and maintenance [52], while an inducible Cre-loxP-mediated inactivation of the β-catenin gene in BM progenitors [53] or a simultaneous β-catenin and γ-catenin deletion did not impair HSCs ability to self-renew and generate all hematopoietic lineages [54].

In Dkk1 transgenic mice [55] or Wnt3a^−/−^ mice [56], Wnt/β-catenin axis impairment blocked hematopoiesis. In contrast to the previous findings, Liu et al. [57] demonstrated that Wnt/β-catenin axis is inhibited in LRP5/6 double deficient mice. This led to a moderate decrease in the adult HSCs pool without causing defects in differentiated cells, which revealed that β-catenin is dispensable during hematopoiesis and lymphopoiesis. A further explanation of these conflictual reports is that, in the hematopoietic compartment, the Wnt/β-catenin pathway could also be modulated by the β-catenin-independent pathway, including cell polarity and other non-canonical Wnt pathways.

Several studies showed that, during hematopoiesis, the expression and modulation of different Wnt proteins, including Wnt3, Wnt5, and Wnt9, seem to have different effects during differentiation stages, which suggests that they are non-redundant [58,59]. For instance, it has been observed that, in the BM niche, Wnt3a played a central role in HSC self-renewal, which preserved HSCs with an immature phenotype, while Wnt5a suppressed proliferation of HSCs, maintaining them in a quiescent state and allowing long-term HSC maintenance [56,60]. Furthermore, it has been demonstrated that Wnt9a regulated in vitro development of human hematopoietic progenitor cells [61].

In general, all these findings suggest that a complex balance of the Wnt/β-catenin pathway is necessary to maintain HSC integrity. Moreover, it has been indicated that the lack of Wnt/β-catenin signaling is detrimental to HSCs function. However, a weak activity of the Wnt/β-catenin pathway is sufficient to restore the HSCs capacity (Figure 3).

## 5. Wnt/β-Catenin Signaling in Leukemia

Aberrant proliferation and differentiation of HSCs is one of the key features of leukemic transformation [62]. Due to the crucial role played by Wnt/β-catenin signaling in hematopoiesis, it is not surprising that deregulation of this signaling network is involved in transformation of healthy HSCs in leukemic stem cells (LSCs) (Figure 3) [63]. LSCs self-renew continuously and are responsible for the maintenance of leukemia cell clones. Uncovering fundamental differences between HSCs and LSCs is a major challenge, which is based on the molecular and phenotypic resemblances between normal and LSCs.

Even though the precise cellular mechanisms underlying leukemic transformation are still unclear, mutations of Wnt ligands or β-catenin-dependent genes have a fundamental role in hematological malignancies pathogenesis, which suggests that these diseases may be “Wnt addicted.”

The aberrant activation of Wnt/β-catenin signaling is related to different hematological malignancies, either by mutations or ectopic activation, including acute myeloid leukemia (AML) [64], chronic myeloid leukemia [65], chronic lymphoid leukemia [66], multiple myeloma [67], and ALL [68]. Moreover, activation of β-catenin is required to sustain AML [69] and ALL drug-resistance [64,70,71,72].

The hyperactivation of the Wnt/β-catenin pathway in ALL may be due to a variety of dysregulated mechanisms, including aberrant expression of Wnt proteins [73], epigenetic alterations [74], activating mutations in β-catenin or inactivating mutations in APC or Axin [75,76], and alterations of the balance of the TCF/LEF complex [77,78].

### 5.1. Wnt/β-Catenin in T-ALL

Wnt/β-catenin pathway deregulation is a frequent event in T-ALL pathogenesis. About 80% of pediatric T-ALL patients showed higher levels of β-catenin compared to healthy controls, which leads to aberrant activation of β-catenin-dependent genes, including *C-MYC*, *BIRC5*, *TCF1*, and *LEF* [79,80]. It is well established that c-myc is an oncogene involved in cancer initiation and progression in different types of tumors [81,82]. Of note, *C-MYC* represents a target gene of Wnt/β-catenin and Notch signaling cascades and it is a promising target for eradicating LSCs in T-ALL [5,83]. Survivin, which is the product of *BIRC5* gene, is downstream of c-myc and its aberrant expression has been observed in ALL primary cells [72].

It has been shown that β-catenin overexpression targeted the highly proliferative CD4^+^ CD8^+^ double positive thymocytes that predispose it to malignant transformation, which leads to a c-myc aberrant activation and develops a Notch-independent leukemia form [84,85]. It has been demonstrated that the Wnt/β-catenin pathway is activated in LSCs of mouse and human T-ALL, and contributes to the drug resistance. The deletion of β-catenin impaired the frequency of LSCs [85,86]. In particular, the activation of the Wnt/β-catenin pathway is typical of minor subpopulations of the leukemic cells where the hypoxia-inducible factor 1α (Hif 1α) is activated [86]. Notably, upregulated β-catenin and Hif1α may sustain LSCs, while deletion of these proteins strongly decreased LSCs frequency, without interfering with the growth of bulk cancer cells [86]. This functional dependency of LSCs may have an important clinical impact on the treatment of T-ALL, as it suggests that inhibition of the Wnt/β-catenin signaling pathway could be a useful approach to treat leukemia, especially for refractory T-ALL patients [86].

Furthermore, *PTEN* deletions cooperated with β-catenin in leukemia development, which demonstrated that activation of the Wnt/β-catenin pathway is related to a subtype of Notch-independent T-ALL, characterized by *C-MYC* rearrangements and *PTEN* mutations [87].

LEF1, which is a member of the LEF/TCF complex, may act as either a tumor suppressor or an oncogene in different cellular contexts. On one side, a significant increase of LEF1 has been observed in more than 25% of adult T-ALL samples [88] and, in TCF1 knockout mice, an increase in LEF1 expression correlated with a T-ALL higher incidence [76]. Of note, a LEF1 increase may be associated with a reduced expression of TCF1 [76]. On the other side, it has been demonstrated that TCF1 may act as a tumor suppressor in T-lymphocytes and LEF1 may be deleted and mutated in T-ALL [77,89]. In addition, loss of TCF1 as a repressor of LEF1 led to increased Wnt activity and could represent the initiating event in lymphoma development [77]. A possible explanation for these contradictory reports could be that TCF1 may act as a tumor suppressor in certain subgroups of pediatric T-ALL [including Early T-cell precursor (ETP)-ALL and myocyte enhancer factor 2C (MEF2C) positive T-ALL] and as an oncogene in other groups of T-ALL.

The role played by the Wnt/β-catenin network in T-ALL pathogenesis is very complex and the precise mechanisms underlying leukemia development remain to be elucidated.

### 5.2. Wnt/β-Catenin in B-ALL

Dysregulation of the Wnt/β-catenin pathway is involved in B-ALL development as demonstrated in a pre-B ALL subtype characterized by (1;19) translocation, where Wnt16b was hyper-activated by the expression of the aberrant E2A-Pbx1 fusion protein. This contributes to leukemia development [90]. Subsequently, Mazieres et al. demonstrated, in t(1;19)-containing cell lines, that Wnt16b was upregulated together with β-catenin, Dvl2, and TCF4, which confirms the activation of the Wnt/β-catenin cascade in these cells. Moreover, they showed that inhibition of Wnt16b induced apoptosis [91]. Wnt3a caused β-catenin accumulation in both B-ALL cell lines and primary samples, without affecting cell survival and proliferation of B-ALL cells [78].

It has also been shown that Wnt proteins increased proliferation of B-ALL cell lines [68] and that the Wnt/β-catenin signaling was important to support B-ALL LSCs survival together with BM stromal cells [92]. Inhibition of the Wnt/β-catenin pathway sensitized ALL cells to cytarabine treatment in vitro and in vivo by overcoming drug resistance in human primary ALL cells and in in vivo experiments.

Therefore, activation of the Wnt/β-catenin network may be a general phenomenon that characterizes B-ALL, acting as a mean for the microenvironment to sustain the survival of the LSCs.

Nygren et al. [93] suggested that the effects of Wnt/β-catenin pathway in B-ALL development could be related to different anomalies such as genetic aberrations, including translocations, or could be due to interactions with other non-canonical signaling pathways, or to β-catenin localization. They demonstrated that aberrant β-catenin localized to the cell membrane alongside N-cadherin. In this context, it is important to emphasize that the pool of β-catenin bound to cadherins at the cell-cell junctions does not mediate the Wnt/β-catenin pathway.

It has been reported that Wnt5a, which promotes GSK3β-independent β-catenin degradation [94], has an anti-proliferative effect in B-ALL cells [95,96]. Wnt5a could negatively regulate the Wnt/β-catenin signaling cascade by inhibiting TCF-mediated transcription, and, thereby, preventing inappropriate activation of the pathway.

It has been demonstrated that mice overexpressing a constitutive active LEF1 mutant developed B-ALL [97]. This observation identified LEF1 as an oncogene, and may have a clinical impact, since LEF1 was found overexpressed in more than 25% of cases of B-ALL in a large cohort of adult patients, and represented an independent adverse prognostic factor [98].

Last, but not least, the Wnt/β-catenin network is dysregulated in Philadelphia positive (Ph^+^) B-ALL, which is a leukemia subset characterized by an extremely poor outcome [99]. In Ph^+^ B-ALL, the over-activation of Wnt/β-catenin pathway in B-ALL may be caused by epigenetic alterations, including hypermethylation of promoters of the Wnt/β-catenin antagonists SFRP, WIF1, and Dkk3 [100]. In particular, the hypermethylation is related to aberrant activation of Wnt/β-catenin network in ALL patients, as documented by the upregulation of Wnt16, Fzd3, TCF1, LEF1, and cyclin D1, as well as by the nuclear localization of β-catenin. Moreover, interactions with BM stromal cells [101], and high expression of a cystic fibrosis transmembrane conductance regulator (CFTR) [102]. CFTR is an ion channel transporter of Cl^−^ and HCO_3_^−^ [102]. However, it was demonstrated that CFTR and protein phosphatase (PP) 2A interact in the cytosol, and, thereby, result in lower PP2A activity and an upregulation of the Wnt/β-catenin signaling network, since PP2A promotes β-catenin degradation by removing an inhibitory phosphorylation on GSK3β [103].

## 6. Targeting Wnt/β-Catenin Signaling in ALL

It has been demonstrated that, in ALL, Wnt/β-catenin antagonism has a role in depleting the self-renewal of drug-resistant LSCs [104,105]. Inhibition of the CBP/β-catenin interaction may push LSCs toward symmetric differentiation, overcoming chemo-resistance, without interfering with the normal HSCs asymmetric differentiation [104,105]. Moreover, in vitro down-regulation of *BIRC5* gene, which encodes survivin, leads to increased chemo-sensitivity, and a clinical trial targeting *BIRC5* with a novel survivin messenger ribonucleic acid (mRNA) antagonist was developed for pediatric patients with relapses of B-ALL (NCT01186328) [106].

Different ways to inhibit Wnt/β-catenin signaling are currently under investigation for acute leukemias (see Table 1).

Several Wnt/β-catenin modulators are now undergoing clinical trials to treat different tumors, including ovarian cancer (NCT02092363), pancreatic cancer (NCT01764477), and advanced solid tumors (NCT02521844). These studies have shown some promising outcomes [108].

In B-ALL cells, it has been observed that the interaction between Wnt/β-catenin pathway and BM stromal cells contributed to cytarabine resistance [92]. Treatment with the tankyrase inhibitor XAV939, which stimulates β-catenin degradation by inhibiting tankyrase, was able to lower chemo-resistance in in vitro and in vivo, most likely by disrupting the BM niche protective support [92].

When added to classical chemotherapeutic drugs, ICG-001, which is a CBP/β-catenin transcription inhibitor, led to decreased self-renewal capacity of ALL cells, downregulated *BIRC5*, and abrogated drug-resistance in primary leukemia cells [72]. Authors demonstrated that ALL relapse patients displayed CBP mutations in about 18.3% of relapse cases and patients that do not relapse [72]. Therefore, CBP mutations can be associated with a worse outcome.

These findings have been confirmed through the inhibition of Wnt by iCRT14 (an inhibitor of β-catenin-dependent transcription) that caused marked cytotoxic effects in ALL cell lines and relapsed ALL samples and restored chemo-sensitivity [71].

Moreover, PKF115-584 (that disrupts the interactions between β-catenin and LEF1, leading to transcriptional inactivation of LEF1) prevented and partially reverted leukemogenesis, by inducing apoptosis and reducing proliferation in human T-ALL cells [81].

In the last few years, crosstalk and correlation between up-regulation of Wnt/β-catenin and multiple signaling pathways have emerged in ALL.

Inhibition of Wnt/β-catenin and phosphatidylinositol 3-kinase (PI3K)/Akt/mechanistic target of rapamycin (mTOR) pathways is considered a possible innovative therapeutic strategy for cancer treatment [109,110].

Independent studies have identified that both PI3K/Akt/mTOR and Wnt/β-catenin signaling networks contribute to leukemia by sustaining neoplastic cell proliferation and drug-resistance [111,112,113]. It has been demonstrated that phosphatase of regenerating liver-3 (PRL-3) high AML cells are dependent on the PI3K/AKT/mTOR and Wnt/β-catenin signaling pathways for survival [114]. Therefore, inhibition of these signaling cascades could achieve robust clinical efficacy for this subtype of AML patients with high PRL-3 expression [114].

Our group recently showed that co-targeting Wnt/β-catenin and PI3K/Akt/mTOR pathways could be a potentially promising treatment in T-ALL settings [115]. Inhibition of the previously mentioned signaling cascades was synergistically cytotoxic to T-ALL cells, especially under hypoxic conditions, that mimic the hypoxic BM microenvironment, where LSCs reside.

Wnt/β-catenin and Notch pathways are linked each other both in development and oncogenesis [116]. Notch1-related leukemogenesis is also dependent on the levels of β-catenin and inhibition of β-catenin compromised survival and proliferation of human T-ALL cell lines carrying activated Notch1 [81].

Another paper demonstrated that *PTEN* loss is frequently associated with Wnt/β-catenin dysregulation in leukemia settings [87]. β-catenin activation, c-myc overexpression, and *PTEN* deletion clustered together in a mouse model with Notch-independent T-ALL [87].

In addition, the aberrant activation of the mitogen-activated protein kinase (MAPK) pathway, which is a common event in cancer, has been shown to be associated with the activation of the Wnt/β-catenin network in relapsed ALL [117].

The bromodomains (BRD) extra terminal proteins (BET) inhibitors directly target BRD proteins, while downregulating c-myc transcription. Given the pivotal role of c-myc in cancer, the effects of BET inhibitors in leukemia were described in several studies and these drugs represent a new therapeutic option. Several BET inhibitors are currently being evaluated in clinical trials in a range of diseases, including hematological malignancies (NCT02158858), even if molecular and cellular mechanisms that govern sensitivity and chemo-resistance remain unknown.

Recently, it has been shown that c-myc inhibition reduced LSCs in mice by demonstrating the involvement of c-myc in LSCs maintenance in ALL [118,119]. Treatment with BET inhibitors reduced c-myc expression and inhibited the growth of relapsed T-ALL samples in vitro and c-myc abrogation depleted LSCs and, consequently, prolonged survival in mice. The pre-clinical efficacy of BET inhibitors has been demonstrated in AML [120].

Combined treatment with a β-catenin inhibitor and a BET inhibitor had strong cytotoxic effects in in vivo and in vitro post-myeloproliferative neoplasms (MPN) AML blast progenitor cells (BPCs) [121,122]. Moreover, in mixed lineage leukemia (MLL)-AF9-driven mouse AML and in human AML cells, resistance to BET inhibitors was due to increased activity of β-catenin and restoration of c-myc expression [123]. BET inhibitor resistance was also shown to emerge from LSCs both in vivo and in vivo and Wnt/β-catenin pathways were significantly upregulated in resistant cells [122,123].

Inhibition of Wnt/β-catenin cascade resulted in restoration of sensitivity to BET inhibitors both in vitro and in vivo [122], which further highlights the crucial influence of this pathway on BET inhibitors efficacy.

All these data together provide new insights into the combination of BET and Wnt/β-catenin inhibitors, which represents a potential new therapeutic strategy to overcome chemo-resistance in leukemia.

Although a large number of compounds inhibiting Wnt/β-catenin signaling have been explored as anti-cancer therapeutics, only two have been tested in clinical studies for hematological disorders. CWP232291 is a β-catenin inhibitor that interferes with the β-catenin-dependent gene expression and is currently being tested for AML (NCT01398462). PRI-724, an ICG-001-derived compound, has entered early-phase clinical trials for hematological malignancies, where it displayed a tolerable toxicity profile (NCT01606579, NCT02195440).

## 7. Conclusions and Perspectives

ALL is an aggressive blood disorder that needs more effective and less toxic targeted therapies, especially for primary resistant and relapsed patients. Chemo-resistance is due to persistence of residual LSCs, which are characterized by quiescence that allow them to escape chemotherapy-induced cell death. Increasing attention has been focused to eradicate the bulk of LSCs by developing novel strategies targeting aberrant signaling networks that lead to LSCs survival and to drug-resistance.

Wnt/β-catenin axis has a central but controversial role in hematopoiesis, which suggests the possible existence of unidentified mechanisms through which the Wnt/β-catenin pathway regulates blood cell production. Moreover, overexpression of Wnt/β-catenin signaling axis has been reported in several cancers, including hematological malignancies and contributes to chemo-resistance. In this context, inhibition of the Wnt/β-catenin pathway offers alternative and interesting possibilities for blood disorders therapeutic intervention, including ALL.

There is now considerable proof that targeting the Wnt/β-catenin network at the level of the receptor/ligand is an attractive therapeutic strategy for inhibiting tumor growth, chemo-resistance, and potentially invasiveness. In particular, the development of Wnt/β-catenin inhibitors at the level of the ligand/receptor, including Wnt secretion inhibitors or antibodies blocking Fzd receptors, are now under investigation and have entered clinical trials.

However, further investigations on the mechanisms underlying the controversial functions of the Wnt/β-catenin pathway will provide new insights in better understanding the processes controlled by Wnt/β-catenin, by identifying those that may safely benefit from administration of selective drugs. Moreover, these studies could provide insight toward other molecules, controlled by the Wnt/β-catenin signaling axis, which would serve as targets for therapeutic intervention.

Another critical step will be to shed light on the reciprocal interactions between Wnt/β-catenin and the BM microenvironment that support the LSCs’ survival. Genetic and pharmacological approaches showed that inhibition of Wnt secretion had no effects on maintenance, self-renewal, and differentiation of adult HSCs [124]. These data could have a clinical relevance, especially for patients displaying high levels of Wnt/β-catenin activation because Wnt/β-catenin inhibitors drugs might not affect healthy HSCs and blood cells production.

Importantly, in line with this finding, a recent study showed that LSCs express high levels of the long isoform of LEF1 compared with immature and more quiescent HSCs. This differential expression of the LEF isoform in LSC versus normal HSCs offers the opportunity to preferentially target LSCs by impairing LEF1-β-catenin interaction, and targeting Wnt/β-catenin axis. This differential dependency of LSC versus HSCs on LEF1-mediated Wnt/β-catenin signaling could potentially be exploited for targeting LSCs while sparing the healthy HSCs [125].

Considering the clinical implications of all these findings, the involvement of Wnt/β-catenin signaling in normal and malignant hematopoiesis should be better addressed.

## Figures and Tables

**Table 1:** Wnt/β-catenin inhibitors used in acute lymphoblastic leukemia (ALL) setting and in clinical trials.

Compound	Target	Clinical Trials	Reference
XAV939	Tankyrase		[92]
ICG-001	CBP/β-catenin		[72]
iCART14	β-catenin-dep gene expression		[71]
PKF115-584	β-catenin/LEF1 interaction		[81]
CWP23229	β-catenin-dep gene expression	NCT01398462	[107]
PRI-724	CBP/β-catenin	NCT01606579 NCT02195440	[72]

## Abbreviations

ALL	acute lymphoblastic leukemia
AML	acute myeloid leukemia
APC	adenomatous polyposis coli
APCDD	adenomatosis polyposis down-regulated
B-ALL	B-acute lymphoblastic leukemia
β-TrC	β-transducin repeats-containing protein
BCL	B-cell lymphoma
BET	bromodomains extra terminal proteins
BM	bone marrow
BRDs	bromodomains
BRG1	ATP-dependent helicase Brahma-related gene
CBP	cyclic adenosine mono phosphate response element -binding protein
CFTR	cystic fibrosis transmembrane conductance regulator
CK	casein kinase
CREB	cyclic adenosine mono phosphate response element
CtBP	C-terminal binding protein
Dkk	dickkopf
Dvl	disheveled
ETP	early T-cell precursor
Fzd	frizzled
GRG/TLE	groucho/transducin-like enhancer
GSK	glycogen synthase kinase
HDAC	histone deacetylase
Hif	hypoxia-inducible factor
HSC	hematopoietic stem cells
LEF	lymphoid enhancing factor
LGR	leucine-rich repeat-containing G-protein coupled receptor
LRP	low-density lipoprotein receptor-related protein
LSC	leukemic stem cells
MAPK	mitogen-activated protein kinase
MEF	myocyte enhancer factor
MLL	mixed lineage leukemia
mTOR	mechanistic target of rapamycin
Ph+	Philadelphia positive
PI3K	phosphatidylinositol 3-kinase
PP	protein phosphatase
PRL-3	phosphatase of regenerating liver-3
RNF43	ring finger 43
SFRP	secreted frizzled-related proteins
SOST	sclerostin
SOSTDC	sclerostin domain containing
T-ALL	T-acute lymphoblastic leukemia
TCF	T-cell factor
WIF	Wnt inhibitory factor
ZNRF3	cell-surface transmembrane E3 ubiquitin ligase zinc and ring finger 3

## References

[1] Siegel R., Naishadham D., Jemal A. (2012). Cancer statistics, 2012. CA Cancer J. Clin..

[2] Hunger S.P., Mullighan C.G. (2015). Acute Lymphoblastic Leukemia in Children. N. Engl. J. Med..

[3] Faderl S., O’Brien S., Pui C.H., Stock W., Wetzler M., Hoelzer D., Kantarjian H.M. (2010). Adult acute lymphoblastic leukemia: Concepts and strategies. Cancer.

[4] Vadillo E., Dorantes-Acosta E., Pelayo R., Schnoor M. (2018). T cell acute lymphoblastic leukemia (T-ALL): New insights into the cellular origins and infiltration mechanisms common and unique among hematologic malignancies. Blood Rev..

[5] Evangelisti C., Chiarini F., McCubrey J.A., Martelli A.M. (2018). Therapeutic Targeting of mTOR in T-Cell Acute Lymphoblastic Leukemia: An Update. Int. J. Mol. Sci..

[6] Iacobucci I., Mullighan C.G. (2017). Genetic Basis of Acute Lymphoblastic Leukemia. J. Clin. Oncol..

[7] Staal F.J., Sen J.M. (2008). The canonical Wnt signaling pathway plays an important role in lymphopoiesis and hematopoiesis. Eur. J. Immunol..

[8] Clevers H., Nusse R. (2012). Wnt/beta-catenin signaling and disease. Cell.

[9] Niehrs C. (2012). The complex world of WNT receptor signalling. Nat. Rev. Mol. Cell Biol..

[10] Stamos J.L., Weis W.I. (2013). The beta-catenin destruction complex. Cold Spring Harb. Perspect. Biol..

[11] Anastas J.N., Moon R.T. (2013). WNT signalling pathways as therapeutic targets in cancer. Nat. Rev. Cancer.

[12] Bienz M. (2014). Signalosome assembly by domains undergoing dynamic head-to-tail polymerization. Trends Biochem. Sci..

[13] Wu G., Huang H., Garcia Abreu J., He X. (2009). Inhibition of GSK3 phosphorylation of beta-catenin via phosphorylated PPPSPXS motifs of Wnt coreceptor LRP6. PLoS ONE.

[14] Stamos J.L., Chu M.L., Enos M.D., Shah N., Weis W.I. (2014). Structural basis of GSK-3 inhibition by N-terminal phosphorylation and by the Wnt receptor LRP6. eLife.

[15] Fiedler M., Graeb M., Mieszczanek J., Rutherford T.J., Johnson C.M., Bienz M. (2015). An ancient Pygo-dependent Wnt enhanceosome integrated by Chip/LDB-SSDP. eLife.

[16] Takemaru K.I., Moon R.T. (2000). The transcriptional coactivator CBP interacts with beta-catenin to activate gene expression. J. Cell Biol..

[17] Shtutman M., Zhurinsky J., Simcha I., Albanese C., D’Amico M., Pestell R., Ben-Ze’ev A. (1999). The cyclin D1 gene is a target of the beta-catenin/LEF-1 pathway. Proc. Natl. Acad. Sci. USA.

[18] He T.C., Sparks A.B., Rago C., Hermeking H., Zawel L., da Costa L.T., Morin P.J., Vogelstein B., Kinzler K.W. (1998). Identification of c-MYC as a target of the APC pathway. Science.

[19] Kim K.A., Kakitani M., Zhao J., Oshima T., Tang T., Binnerts M., Liu Y., Boyle B., Park E., Emtage P. (2005). Mitogenic influence of human R-spondin1 on the intestinal epithelium. Science.

[20] Carmon K.S., Gong X., Lin Q., Thomas A., Liu Q. (2011). R-spondins function as ligands of the orphan receptors LGR4 and LGR5 to regulate Wnt/beta-catenin signaling. Proc. Natl. Acad. Sci. USA.

[21] Glinka A., Dolde C., Kirsch N., Huang Y.L., Kazanskaya O., Ingelfinger D., Boutros M., Cruciat C.M., Niehrs C. (2011). LGR4 and LGR5 are R-spondin receptors mediating Wnt/beta-catenin and Wnt/PCP signalling. EMBO Rep..

[22] Hao H.X., Xie Y., Zhang Y., Charlat O., Oster E., Avello M., Lei H., Mickanin C., Liu D., Ruffner H. (2012). ZNRF3 promotes Wnt receptor turnover in an R-spondin-sensitive manner. Nature.

[23] Koo B.K., Spit M., Jordens I., Low T.Y., Stange D.E., van de Wetering M., van Es J.H., Mohammed S., Heck A.J., Maurice M.M. (2012). Tumour suppressor RNF43 is a stem-cell E3 ligase that induces endocytosis of Wnt receptors. Nature.

[24] Jiang X., Hao H.X., Growney J.D., Woolfenden S., Bottiglio C., Ng N., Lu B., Hsieh M.H., Bagdasarian L., Meyer R. (2013). Inactivating mutations of RNF43 confer Wnt dependency in pancreatic ductal adenocarcinoma. Proc. Natl. Acad. Sci. USA.

[25] Hao H.X., Jiang X., Cong F. (2016). Control of Wnt Receptor Turnover by R-spondin-ZNRF3/RNF43 Signaling Module and Its Dysregulation in Cancer. Cancers.

[26] Giannakis M., Hodis E., Jasmine Mu X., Yamauchi M., Rosenbluh J., Cibulskis K., Saksena G., Lawrence M.S., Qian Z.R., Nishihara R. (2014). RNF43 is frequently mutated in colorectal and endometrial cancers. Nat. Genet..

[27] Seshagiri S., Stawiski E.W., Durinck S., Modrusan Z., Storm E.E., Conboy C.B., Chaudhuri S., Guan Y., Janakiraman V., Jaiswal B.S. (2012). Recurrent R-spondin fusions in colon cancer. Nature.

[28] Braunger B.M., Tamm E.R. (2012). The different functions of Norrin. Adv. Exp. Med. Biol..

[29] Ke J., Harikumar K.G., Erice C., Chen C., Gu X., Wang L., Parker N., Cheng Z., Xu W., Williams B.O. (2013). Structure and function of Norrin in assembly and activation of a Frizzled 4-Lrp5/6 complex. Genes Dev..

[30] Niehrs C. (2004). Norrin and frizzled: A new vein for the eye. Dev. Cell.

[31] Cruciat C.M., Niehrs C. (2013). Secreted and transmembrane wnt inhibitors and activators. Cold Spring Harb. Perspect. Biol..

[32] Shimomura Y., Agalliu D., Vonica A., Luria V., Wajid M., Baumer A., Belli S., Petukhova L., Schinzel A., Brivanlou A.H. (2010). APCDD1 is a novel Wnt inhibitor mutated in hereditary hypotrichosis simplex. Nature.

[33] Ahn Y., Sanderson B.W., Klein O.D., Krumlauf R. (2010). Inhibition of Wnt signaling by Wise (Sostdc1) and negative feedback from Shh controls tooth number and patterning. Development.

[34] Semenov M., Tamai K., He X. (2005). SOST is a ligand for LRP5/LRP6 and a Wnt signaling inhibitor. J. Biol. Chem..

[35] Li X., Zhang Y., Kang H., Liu W., Liu P., Zhang J., Harris S.E., Wu D. (2005). Sclerostin binds to LRP5/6 and antagonizes canonical Wnt signaling. J. Biol. Chem..

[36] Sekiya T., Zaret K.S. (2007). Repression by Groucho/TLE/Grg proteins: Genomic site recruitment generates compacted chromatin in vitro and impairs activator binding in vivo. Mol. Cell..

[37] Shooshtarizadeh P., Helness A., Vadnais C., Brouwer N., Beauchemin H., Chen R., Bagci H., Staal F.J.T., Cote J.F., Moroy T. (2019). Gfi1b regulates the level of Wnt/beta-catenin signaling in hematopoietic stem cells and megakaryocytes. Nat. Commun..

[38] Staal F.J., Luis T.C., Tiemessen M.M. (2008). WNT signalling in the immune system: WNT is spreading its wings. Nat. Rev. Immunol..

[39] Mao B., Wu W., Li Y., Hoppe D., Stannek P., Glinka A., Niehrs C. (2001). LDL-receptor-related protein 6 is a receptor for Dickkopf proteins. Nature.

[40] Wu W., Glinka A., Delius H., Niehrs C. (2000). Mutual antagonism between dickkopf1 and dickkopf2 regulates Wnt/beta-catenin signalling. Curr. Biol..

[41] Li L., Mao J., Sun L., Liu W., Wu D. (2002). Second cysteine-rich domain of Dickkopf-2 activates canonical Wnt signaling pathway via LRP-6 independently of dishevelled. J. Biol. Chem..

[42] Mao B., Niehrs C. (2003). Kremen2 modulates Dickkopf2 activity during Wnt/LRP6 signaling. Gene.

[43] Brott B.K., Sokol S.Y. (2002). Regulation of Wnt/LRP signaling by distinct domains of Dickkopf proteins. Mol. Cell Biol..

[44] Jiang X., Charlat O., Zamponi R., Yang Y., Cong F. (2015). Dishevelled promotes Wnt receptor degradation through recruitment of ZNRF3/RNF43 E3 ubiquitin ligases. Mol. Cell.

[45] Chiarini F., Lonetti A., Evangelisti C., Buontempo F., Orsini E., Cappellini A., Neri L.M., McCubrey J.A., Martelli A.M. (2016). Advances in understanding the acute lymphoblastic leukemia bone marrow microenvironment: From biology to therapeutic targeting. Biochim. Biophys. Acta.

[46] Luis T.C., Ichii M., Brugman M.H., Kincade P., Staal F.J. (2012). Wnt signaling strength regulates normal hematopoiesis and its deregulation is involved in leukemia development. Leukemia.

[47] Reya T., Duncan A.W., Ailles L., Domen J., Scherer D.C., Willert K., Hintz L., Nusse R., Weissman I.L. (2003). A role for Wnt signalling in self-renewal of haematopoietic stem cells. Nature.

[48] Scheller M., Huelsken J., Rosenbauer F., Taketo M.M., Birchmeier W., Tenen D.G., Leutz A. (2006). Hematopoietic stem cell and multilineage defects generated by constitutive beta-catenin activation. Nat. Immunol..

[49] Kirstetter P., Anderson K., Porse B.T., Jacobsen S.E., Nerlov C. (2006). Activation of the canonical Wnt pathway leads to loss of hematopoietic stem cell repopulation and multilineage differentiation block. Nat. Immunol..

[50] Luis T.C., Naber B.A., Roozen P.P., Brugman M.H., de Haas E.F., Ghazvini M., Fibbe W.E., van Dongen J.J., Fodde R., Staal F.J. (2011). Canonical wnt signaling regulates hematopoiesis in a dosage-dependent fashion. Cell Stem Cell.

[51] Famili F., Brugman M.H., Taskesen E., Naber B.E.A., Fodde R., Staal F.J.T. (2016). High Levels of Canonical Wnt Signaling Lead to Loss of Stemness and Increased Differentiation in Hematopoietic Stem Cells. Stem Cell Rep..

[52] Zhao C., Blum J., Chen A., Kwon H.Y., Jung S.H., Cook J.M., Lagoo A., Reya T. (2007). Loss of beta-catenin impairs the renewal of normal and CML stem cells in vivo. Cancer Cell.

[53] Cobas M., Wilson A., Ernst B., Mancini S.J., MacDonald H.R., Kemler R., Radtke F. (2004). Beta-catenin is dispensable for hematopoiesis and lymphopoiesis. J. Exp. Med..

[54] Jeannet G., Scheller M., Scarpellino L., Duboux S., Gardiol N., Back J., Kuttler F., Malanchi I., Birchmeier W., Leutz A. (2008). Long-term, multilineage hematopoiesis occurs in the combined absence of beta-catenin and gamma-catenin. Blood.

[55] Fleming H.E., Janzen V., Lo Celso C., Guo J., Leahy K.M., Kronenberg H.M., Scadden D.T. (2008). Wnt signaling in the niche enforces hematopoietic stem cell quiescence and is necessary to preserve self-renewal in vivo. Cell Stem Cell.

[56] Luis T.C., Weerkamp F., Naber B.A., Baert M.R., de Haas E.F., Nikolic T., Heuvelmans S., De Krijger R.R., van Dongen J.J., Staal F.J. (2009). Wnt3a deficiency irreversibly impairs hematopoietic stem cell self-renewal and leads to defects in progenitor cell differentiation. Blood.

[57] Liu J., Cui Z., Wang F., Yao Y., Yu G., Cao D., Niu S., You M., Sun Z., Lian D. (2019). Lrp5 and Lrp6 are required for maintaining self-renewal and differentiation of hematopoietic stem cells. FASEB J..

[58] Wang Y., Nakayama N. (2009). WNT and BMP signaling are both required for hematopoietic cell development from human ES cells. Stem Cell Res..

[59] Gertow K., Hirst C.E., Yu Q.C., Ng E.S., Pereira L.A., Davis R.P., Stanley E.G., Elefanty A.G. (2013). WNT3A promotes hematopoietic or mesenchymal differentiation from hESCs depending on the time of exposure. Stem Cell Rep..

[60] Luis T.C., Naber B.A., Fibbe W.E., van Dongen J.J., Staal F.J. (2010). Wnt3a nonredundantly controls hematopoietic stem cell function and its deficiency results in complete absence of canonical Wnt signaling. Blood.

[61] Richter J., Stanley E.G., Ng E.S., Elefanty A.G., Traver D., Willert K. (2018). WNT9A Is a Conserved Regulator of Hematopoietic Stem and Progenitor Cell Development. Genes.

[62] Gezer D., Vukovic M., Soga T., Pollard P.J., Kranc K.R. (2014). Concise review: Genetic dissection of hypoxia signaling pathways in normal and leukemic stem cells. Stem Cells.

[63] Lento W., Congdon K., Voermans C., Kritzik M., Reya T. (2013). Wnt signaling in normal and malignant hematopoiesis. Cold Spring Harb. Perspect. Biol..

[64] Wang Y., Krivtsov A.V., Sinha A.U., North T.E., Goessling W., Feng Z., Zon L.I., Armstrong S.A. (2010). The Wnt/beta-catenin pathway is required for the development of leukemia stem cells in AML. Science.

[65] Heidel F.H., Bullinger L., Feng Z., Wang Z., Neff T.A., Stein L., Kalaitzidis D., Lane S.W., Armstrong S.A. (2012). Genetic and pharmacologic inhibition of beta-catenin targets imatinib-resistant leukemia stem cells in CML. Cell Stem Cell.

[66] Lu J., Ma Z., Hsieh J.C., Fan C.W., Chen B., Longgood J.C., Williams N.S., Amatruda J.F., Lum L., Chen C. (2009). Structure-activity relationship studies of small-molecule inhibitors of Wnt response. Bioorg. Med. Chem. Lett..

[67] Derksen P.W., Tjin E., Meijer H.P., Klok M.D., MacGillavry H.D., van Oers M.H., Lokhorst H.M., Bloem A.C., Clevers H., Nusse R. (2004). Illegitimate WNT signaling promotes proliferation of multiple myeloma cells. Proc. Natl. Acad. Sci. USA.

[68] Khan N.I., Bradstock K.F., Bendall L.J. (2007). Activation of Wnt/beta-catenin pathway mediates growth and survival in B-cell progenitor acute lymphoblastic leukaemia. Br. J. Haematol..

[69] Yeung J., Esposito M.T., Gandillet A., Zeisig B.B., Griessinger E., Bonnet D., So C.W. (2010). beta-Catenin mediates the establishment and drug resistance of MLL leukemic stem cells. Cancer Cell.

[70] Hu Y., Chen Y., Douglas L., Li S. (2009). beta-Catenin is essential for survival of leukemic stem cells insensitive to kinase inhibition in mice with BCR-ABL-induced chronic myeloid leukemia. Leukemia.

[71] Dandekar S., Romanos-Sirakis E., Pais F., Bhatla T., Jones C., Bourgeois W., Hunger S.P., Raetz E.A., Hermiston M.L., Dasgupta R. (2014). Wnt inhibition leads to improved chemosensitivity in paediatric acute lymphoblastic leukaemia. Br. J. Haematol..

[72] Gang E.J., Hsieh Y.T., Pham J., Zhao Y., Nguyen C., Huantes S., Park E., Naing K., Klemm L., Swaminathan S. (2014). Small-molecule inhibition of CBP/catenin interactions eliminates drug-resistant clones in acute lymphoblastic leukemia. Oncogene.

[73] Wu Q.L., Zierold C., Ranheim E.A. (2009). Dysregulation of Frizzled 6 is a critical component of B-cell leukemogenesis in a mouse model of chronic lymphocytic leukemia. Blood.

[74] Roman-Gomez J., Jimenez-Velasco A., Cordeu L., Vilas-Zornoza A., San Jose-Eneriz E., Garate L., Castillejo J.A., Martin V., Prosper F., Heiniger A. (2007). WNT5A, a putative tumour suppressor of lymphoid malignancies, is inactivated by aberrant methylation in acute lymphoblastic leukaemia. Eur. J. Cancer.

[75] Ng O.H., Erbilgin Y., Firtina S., Celkan T., Karakas Z., Aydogan G., Turkkan E., Yildirmak Y., Timur C., Zengin E. (2014). Deregulated WNT signaling in childhood T-cell acute lymphoblastic leukemia. Blood Cancer J..

[76] Yu S., Zhou X., Steinke F.C., Liu C., Chen S.C., Zagorodna O., Jing X., Yokota Y., Meyerholz D.K., Mullighan C.G. (2012). The TCF-1 and LEF-1 transcription factors have cooperative and opposing roles in T cell development and malignancy. Immunity.

[77] Tiemessen M.M., Baert M.R., Schonewille T., Brugman M.H., Famili F., Salvatori D.C., Meijerink J.P., Ozbek U., Clevers H., van Dongen J.J. (2012). The nuclear effector of Wnt-signaling, Tcf1, functions as a T-cell-specific tumor suppressor for development of lymphomas. PLoS Biol..

[78] Nygren M.K., Dosen G., Hystad M.E., Stubberud H., Funderud S., Rian E. (2007). Wnt3A activates canonical Wnt signalling in acute lymphoblastic leukaemia (ALL) cells and inhibits the proliferation of B-ALL cell lines. Br. J. Haematol..

[79] Arensman M.D., Telesca D., Lay A.R., Kershaw K.M., Wu N., Donahue T.R., Dawson D.W. (2014). The CREB-binding protein inhibitor ICG-001 suppresses pancreatic cancer growth. Mol. Cancer Ther..

[80] Fernandes J.C., Rodrigues Alves A.P.N., Machado-Neto J.A., Scopim-Ribeiro R., Fenerich B.A., da Silva F.B., Simoes B.P., Rego E.M., Traina F. (2017). IRS1/beta-Catenin Axis Is Activated and Induces MYC Expression in Acute Lymphoblastic Leukemia Cells. J. Cell Biochem..

[81] Gekas C., D’Altri T., Aligue R., Gonzalez J., Espinosa L., Bigas A. (2016). beta-Catenin is required for T-cell leukemia initiation and MYC transcription downstream of Notch1. Leukemia.

[82] Dang C.V. (2012). MYC on the path to cancer. Cell.

[83] Schubbert S., Cardenas A., Chen H., Garcia C., Guo W., Bradner J., Wu H. (2014). Targeting the MYC and PI3K pathways eliminates leukemia-initiating cells in T-cell acute lymphoblastic leukemia. Cancer Res..

[84] Guo Z., Dose M., Kovalovsky D., Chang R., O’Neil J., Look A.T., von Boehmer H., Khazaie K., Gounari F. (2007). Beta-catenin stabilization stalls the transition from double-positive to single-positive stage and predisposes thymocytes to malignant transformation. Blood.

[85] Guo W., Lasky J.L., Chang C.J., Mosessian S., Lewis X., Xiao Y., Yeh J.E., Chen J.Y., Iruela-Arispe M.L., Varella-Garcia M. (2008). Multi-genetic events collaboratively contribute to Pten-null leukaemia stem-cell formation. Nature.

[86] Giambra V., Jenkins C.E., Lam S.H., Hoofd C., Belmonte M., Wang X., Gusscott S., Gracias D., Weng A.P. (2015). Leukemia stem cells in T-ALL require active Hif1alpha and Wnt signaling. Blood.

[87] Kaveri D., Kastner P., Dembele D., Nerlov C., Chan S., Kirstetter P. (2013). beta-Catenin activation synergizes with Pten loss and Myc overexpression in Notch-independent T-ALL. Blood.

[88] Guo X., Zhang R., Liu J., Li M., Song C., Dovat S., Li J., Ge Z. (2015). Characterization of LEF1 High Expression and Novel Mutations in Adult Acute Lymphoblastic Leukemia. PLoS ONE.

[89] Gutierrez A., Sanda T., Ma W., Zhang J., Grebliunaite R., Dahlberg S., Neuberg D., Protopopov A., Winter S.S., Larson R.S. (2010). Inactivation of LEF1 in T-cell acute lymphoblastic leukemia. Blood.

[90] McWhirter J.R., Neuteboom S.T., Wancewicz E.V., Monia B.P., Downing J.R., Murre C. (1999). Oncogenic homeodomain transcription factor E2A-Pbx1 activates a novel WNT gene in pre-B acute lymphoblastoid leukemia. Proc. Natl. Acad. Sci. USA.

[91] Mazieres J., You L., He B., Xu Z., Lee A.Y., Mikami I., McCormick F., Jablons D.M. (2005). Inhibition of Wnt16 in human acute lymphoblastoid leukemia cells containing the t(1;19) translocation induces apoptosis. Oncogene.

[92] Yang Y., Mallampati S., Sun B., Zhang J., Kim S.B., Lee J.S., Gong Y., Cai Z., Sun X. (2013). Wnt pathway contributes to the protection by bone marrow stromal cells of acute lymphoblastic leukemia cells and is a potential therapeutic target. Cancer Lett..

[93] Nygren M.K., Dosen-Dahl G., Stubberud H., Walchli S., Munthe E., Rian E. (2009). beta-catenin is involved in N-cadherin-dependent adhesion, but not in canonical Wnt signaling in E2A-PBX1-positive B acute lymphoblastic leukemia cells. Exp. Hematol..

[94] Topol L., Jiang X., Choi H., Garrett-Beal L., Carolan P.J., Yang Y. (2003). Wnt-5a inhibits the canonical Wnt pathway by promoting GSK-3-independent beta-catenin degradation. J. Cell. Biol..

[95] Liang H., Chen Q., Coles A.H., Anderson S.J., Pihan G., Bradley A., Gerstein R., Jurecic R., Jones S.N. (2003). Wnt5a inhibits B cell proliferation and functions as a tumor suppressor in hematopoietic tissue. Cancer Cell.

[96] Ying J., Li H., Chen Y.W., Srivastava G., Gao Z., Tao Q. (2007). WNT5A is epigenetically silenced in hematologic malignancies and inhibits leukemia cell growth as a tumor suppressor. Blood.

[97] Petropoulos K., Arseni N., Schessl C., Stadler C.R., Rawat V.P., Deshpande A.J., Heilmeier B., Hiddemann W., Quintanilla-Martinez L., Bohlander S.K. (2008). A novel role for Lef-1, a central transcription mediator of Wnt signaling, in leukemogenesis. J. Exp. Med..

[98] Kuhnl A., Gokbuget N., Kaiser M., Schlee C., Stroux A., Burmeister T., Mochmann L.H., Hoelzer D., Hofmann W.K., Thiel E. (2011). Overexpression of LEF1 predicts unfavorable outcome in adult patients with B-precursor acute lymphoblastic leukemia. Blood.

[99] Owattanapanich W., Rujirachun P., Ungprasert P., Buaboonnam J., Techavichit P. (2019). Prevalence and Clinical Outcome of Philadelphia-Like Acute Lymphoblastic Leukemia: Systematic Review and Meta-analysis. Clin. Lymphoma Myeloma Leuk..

[100] Martin V., Agirre X., Jimenez-Velasco A., Jose-Eneriz E.S., Cordeu L., Garate L., Vilas-Zornoza A., Castillejo J.A., Heiniger A., Prosper F. (2008). Methylation status of Wnt signaling pathway genes affects the clinical outcome of Philadelphia-positive acute lymphoblastic leukemia. Cancer Sci..

[101] Zhang C., Zhang X., Yang S.J., Chen X.H. (2017). Growth of tyrosine kinase inhibitor-resistant Philadelphia-positive acute lymphoblastic leukemia: Role of bone marrow stromal cells. Oncol. Lett..

[102] Linsdell P. (2018). Cystic fibrosis transmembrane conductance regulator (CFTR): Making an ion channel out of an active transporter structure. Channels.

[103] Yang X., Yan T., Gong Y., Liu X., Sun H., Xu W., Wang C., Naren D., Zheng Y. (2017). High CFTR expression in Philadelphia chromosome-positive acute leukemia protects and maintains continuous activation of BCR-ABL and related signaling pathways in combination with PP2A. Oncotarget.

[104] Park E., Gang E.J., Hsieh Y.T., Schaefer P., Chae S., Klemm L., Huantes S., Loh M., Conway E.M., Kang E.S. (2011). Targeting survivin overcomes drug resistance in acute lymphoblastic leukemia. Blood.

[105] Morrison D.J., Hogan L.E., Condos G., Bhatla T., Germino N., Moskowitz N.P., Lee L., Bhojwani D., Horton T.M., Belitskaya-Levy I. (2012). Endogenous knockdown of survivin improves chemotherapeutic response in ALL models. Leukemia.

[106] Raetz E.A., Morrison D., Romanos-Sirakis E., Gaynon P., Sposto R., Bhojwani D., Bostrom B.C., Brown P., Eckroth E., Cassar J. (2014). A phase I study of EZN-3042, a novel survivin messenger ribonucleic acid (mRNA) antagonist, administered in combination with chemotherapy in children with relapsed acute lymphoblastic leukemia (ALL): A report from the therapeutic advances in childhood leukemia and lymphoma (TACL) consortium. J. Pediatr. Hematol. Oncol..

[107] Curtin J.C., Lorenzi M.V. (2010). Drug discovery approaches to target Wnt signaling in cancer stem cells. Oncotarget.

[108] Harb J., Lin P.J., Hao J. (2019). Recent Development of Wnt Signaling Pathway Inhibitors for Cancer Therapeutics. Curr. Oncol. Rep..

[109] Arques O., Chicote I., Puig I., Tenbaum S.P., Argiles G., Dienstmann R., Fernandez N., Caratu G., Matito J., Silberschmidt D. (2016). Tankyrase Inhibition Blocks Wnt/beta-Catenin Pathway and Reverts Resistance to PI3K and AKT Inhibitors in the Treatment of Colorectal Cancer. Clin. Cancer Res..

[110] Park Y.L., Kim H.P., Cho Y.W., Min D.W., Cheon S.K., Lim Y.J., Song S.H., Kim S.J., Han S.W., Park K.J. (2019). Activation of WNT/beta-catenin signaling results in resistance to a dual PI3K/mTOR inhibitor in colorectal cancer cells harboring PIK3CA mutations. Int. J. Cancer.

[111] Yang P., Li Z., Wang Y., Zhang L., Wu H. (2015). Secreted pyruvate kinase M2 facilitates cell migration via PI3K/Akt and Wnt/beta-catenin pathway in colon cancer cells. Biochem. Biophys. Res. Commun..

[112] Zhang L.N., Zhao L., Yan X.L., Huang Y.H. (2019). Loss of G3BP1 suppresses proliferation, migration, and invasion of esophageal cancer cells via Wnt/beta-catenin and PI3K/AKT signaling pathways. J. Cell Physiol..

[113] Jefferies M.T., Cox A.C., Shorning B.Y., Meniel V., Griffiths D., Kynaston H.G., Smalley M.J., Clarke A.R. (2017). PTEN loss and activation of K-RAS and beta-catenin cooperate to accelerate prostate tumourigenesis. J. Pathol..

[114] Zhou J., Toh S.H., Chan Z.L., Quah J.Y., Chooi J.Y., Tan T.Z., Chong P.S.Y., Zeng Q., Chng W.J. (2018). A loss-of-function genetic screening reveals synergistic targeting of AKT/mTOR and WTN/beta-catenin pathways for treatment of AML with high PRL-3 phosphatase. J. Hematol. Oncol..

[115] Evangelisti C., Chiarini F., Cappellini A., Paganelli F., Fini M., Santi S., Martelli A.M., Neri L.M. (2020). Targeting Wnt/beta-catenin and PI3K/Akt/mTOR pathways in T-cell acute lymphoblastic leukemia. J. Cell Physiol..

[116] Bigas A., Guiu J., Gama-Norton L. (2013). Notch and Wnt signaling in the emergence of hematopoietic stem cells. Blood Cells Mol. Dis..

[117] Hogan L.E., Meyer J.A., Yang J., Wang J., Wong N., Yang W., Condos G., Hunger S.P., Raetz E., Saffery R. (2011). Integrated genomic analysis of relapsed childhood acute lymphoblastic leukemia reveals therapeutic strategies. Blood.

[118] Roderick J.E., Tesell J., Shultz L.D., Brehm M.A., Greiner D.L., Harris M.H., Silverman L.B., Sallan S.E., Gutierrez A., Look A.T. (2014). c-Myc inhibition prevents leukemia initiation in mice and impairs the growth of relapsed and induction failure pediatric T-ALL cells. Blood.

[119] Loosveld M., Castellano R., Gon S., Goubard A., Crouzet T., Pouyet L., Prebet T., Vey N., Nadel B., Collette Y. (2014). Therapeutic targeting of c-Myc in T-cell acute lymphoblastic leukemia, T-ALL. Oncotarget.

[120] Dawson M.A., Kouzarides T. (2012). Cancer epigenetics: From mechanism to therapy. Cell.

[121] Saenz D.T., Fiskus W., Manshouri T., Mill C.P., Qian Y., Raina K., Rajapakshe K., Coarfa C., Soldi R., Bose P. (2019). Targeting nuclear beta-catenin as therapy for post-myeloproliferative neoplasm secondary AML. Leukemia.

[122] Rathert P., Roth M., Neumann T., Muerdter F., Roe J.S., Muhar M., Deswal S., Cerny-Reiterer S., Peter B., Jude J. (2015). Transcriptional plasticity promotes primary and acquired resistance to BET inhibition. Nature.

[123] Fong C.Y., Gilan O., Lam E.Y., Rubin A.F., Ftouni S., Tyler D., Stanley K., Sinha D., Yeh P., Morison J. (2015). BET inhibitor resistance emerges from leukaemia stem cells. Nature.

[124] Kabiri Z., Numata A., Kawasaki A., Tenen D.G., Virshup D.M. (2015). Wnts are dispensable for differentiation and self-renewal of adult murine hematopoietic stem cells. Blood.

[125] Feder K., Edmaier-Schroger K., Rawat V.P.S., Kirsten N., Metzeler K., Kraus J.M., Dohner K., Dohner H., Kestler H.A., Feuring-Buske M. (2019). Differences in expression and function of LEF1 isoforms in normal versus leukemic hematopoiesis. Leukemia.

